# Malpractice litigation related to management of varicocele: a legal database review

**DOI:** 10.1038/s41443-024-00881-y

**Published:** 2024-04-10

**Authors:** Eric Zhou, Nicholas Sellke, Helen Sun, Kimberly Tay, Sherry Mortach, Ramy Abou Ghayda, Aram Loeb, Nannan Thirumavalavan

**Affiliations:** 1 Urology Institute, University Hospitals Cleveland Health System, Cleveland, OH USA; 2 Case Western Reserve University School of Medicine, Cleveland, OH USA

**Keywords:** Health care, Risk factors

## Introduction

Medical malpractice is a legal definition wherein a medical professional, through either a deliberate act or omission, deviates from the standard of care to cause injury or death to a patient [1]. Malpractice lawsuits are a fairly common occurrence in the United States, and frequency has steadily been increasing since the 1960s [2]. It is estimated that approximately 85,000 cases are filed each year nationwide [3]. Between 2010 and 2019, $42 billion was paid to malpractice victims [3]. A 2010 report estimated that annual malpractice costs, which included settlements, administrative expenses, and defensive medicine, amounted to $55.6 billion in 2008 dollars, equivalent to $79.5 billion in 2023 [4]. Medical malpractice plays a large role in healthcare costs and considerations in the United States.

From 2017 to 2021, the most common claims revolved around improper treatment (28.5%), failure to diagnose or inappropriate diagnosis (26%), and surgery (24%) [3]. On average, 7.4% of physicians annually have a claim filed against them [5]. Urologists have more claims compared to the average, ranking eighth out of 25 specialties, and would be sued at least twice over the course of their career [5, 6]. A policy research study from the American Medical Association in 2023 found that 38.3% of urologists had been sued at least once in their career [7]. These claims can have significant impacts on the physicians involved, both emotionally and in productivity.

There have been studies that examined litigation procedures stemming from the management and treatment of various urological issues [6, 8]. Varicocele is a common urologic condition and can be associated with pain, subfertility, and infertility, thus greatly impacting patient well-being and quality of life. It has a prevalence of 15% in the general male population and up to 35% in males with infertility [9]. Varicocele has a range of treatment options, from observation to pain management to surgical correction. To our knowledge, no legal claims database investigation into varicocele cases exists. Elucidating the factors that go into varicocele litigation could provide valuable insight into management considerations for this disease and provide an opportunity to improve care for patients. This study sought to investigate factors associated with malpractice litigation surrounding varicocele management.

## Methods

The Casetext online legal database was queried for cases with the terms: “Malpractice and (Varicocele or Varicocelectomy).” Casetext maintains a database that provides state and federal case summaries and transcripts. It covers all 50 state and federal cases, statutes, regulations, and rules. Because the cases in Casetext are publicly available, our study did not need Institutional Review Board review.

Cases that included varicocele management and with a final judgment were included. We excluded cases in which varicocele management was not the medical issue being discussed. We documented the following variables for each case: date of case, whether a urologist was involved in the defense, whether the plaintiff was incarcerated, alleged breaches of duty, alleged damages, and legal outcome. Data were analyzed using descriptive characteristics.

## Results

Our database search yielded a total of 26 cases, of which 10 met the inclusion criteria. Table 1 summarizes each of the 10 cases. The cases occurred between the years 1931 and 2022, with 60% (6/10) having happened since 2013. 60% (6/10) of the cases involved an inmate as the plaintiff. The most common alleged breach of duty was violation of the 8th Amendment (50%, 5/10), which, among other measures, protects against cruel and unusual punishment. All those cases involved inmates. The other alleged breaches included negligence or indifference to the patient’s medical condition (40%, 4/10) and disability discrimination (10%, 1/10). Regarding the overall nature of the breach of duty, half of the cases cited an alleged lack of treatment or inappropriate treatment (e.g., prescribing medication when surgery should have occurred) while the other half cited consequences of surgery (e.g., failure to correct the varicocele, failure to perform operation according to medical standards, infection resulting from the operation).

**Table 1:** Summary of cases analyzed in the study, including the name of the case and year, summary of the case, alleged breaches of duty, alleged damages, legal outcome, and summary of the outcome.

Case title	Case summary	Alleged breach(es) of duty	Alleged damage(s)	Legal outcome	Outcome summary
Nicholas v. Jacobson (1931)	Plaintiff sued for damages for alleged malpractice. The plaintiff had a left varicocele and an operation was performed to remove the varicose veins. The postoperative wound became infected and was treated by the defendant. Several months later, the plaintiff discovered that his left testicle was gone and claims that the operation caused this.	Deviation from standard of care, malpractice and negligence leading to loss of left testicle	Loss of testicle	Favoring Defense (includes urologist)	Not enough evidence to demonstrate malpractice. Expert testimony could not say post facto that the defendant did not act in accord to sound medical judgment and skill. The loss of the testicle could have been attributed to a number of different causes.
Champion v. Bennetts (1951)	The plaintiff was diagnosed with varicocele by defendant, and subsequently underwent an operation. Plaintiff alleges that the defendant exhibited negligence during the operation due to a retained “rubber tube” within the scrotum. This caused severe complications that required removal of the plaintiff’s testicle.	Negligence, deviation from standard of care resulting in damage	Severe pain, infection, loss of testicle and subsequent shortening of life expectancy and sterility. Damages were also sought for loss of earnings during the incapacitation period.	Favoring plaintiff (Defense was a urologist)	Jury decided that the defendant did not follow standard procedure, thus demonstrating negligence.
Cohen v. Cabrini Medical Center (1999)	Plaintiff is the wife of a patient who was treated for varicocele. She had assumed that the treatment would have allowed them to conceive without in vitro fertilization.	Malpractice	Loss of offspring, continued inability to conceive	Favoring defense (includes urologist)	The varicocelectomy does not guarantee ability to conceive. The plaintiff was never given assurances that the procedure would increase fertility. The claim was dismissed as being too speculative to be compensable.
Abdur-Rahiim v. Doe (2009)	Plaintiff (a prisoner) suffered from a varicocele and had surgery. He alleges the surgery did not alleviate his pain although he was told that it would	Negligence, violation of 8^th^ Amendment rights against cruel and unusual punishment	Continued pain	Favoring defense (includes urologist)	The plaintiff’s complaint was time-barred, and he did not properly exhaust his administrative remedies before filing the suit.
Diaz v. Spencer (2013)	Plaintiff (a prisoner) had surgery for a varicocele and contents that the surgery was not successful. Claims he continued to suffer pain and alleges there was concurrent negligence and malpractice regarding treatment for the pain	Medical indifference and malpractice	Prolonged pain	Favoring defense (includes urologist)	Court states the plaintiff’s claims are not entirely intelligible as he did not set forth the underlying facts to support his claims of negligence, deliberate indifference, and the “who, what, when, where, and why” information necessary to state plausible claims.
Canales v. Abramson (2014)	Plaintiff (a prisoner) saw defendant for pain in penis and scrotum. Was later diagnosed with varicocele among other issues, but was only given pain medication. The plaintiff claims the defense conspired against him to deny him proper care.	Violation of 8th amendment (cruel and unusual punishment) when defendants were deliberately indifferent to his serious medical needs relating to his pain.	Prolonged pain	Favoring defense (includes a urologist)	Court ruled there was insufficient evidence that the defendants deliberately withheld medical treatment and/or purposely denied him medical care. Also ruled that a difference in medical opinion over treatment course between medical staff and prisoner does not constitute cruel and unusual punishment.
Ingram v. Warden (2015)	The plaintiff (a prisoner) had varicoceles and alleges that the defendants denied him medical treatment, leading to loss of a testicle	Violation of 8th amendment rights	Pain and loss of testicle	Favoring defense (Warden, i.e., Director of Baltimore County Detention Center; and Conmed Healthcare Management, Inc.	The claim does not give sufficient evidence that the defendants acted with deliberate indifference to the plaintiff’s complaints.
Johnson v Estate of Obaisi (2019)	Plaintiff (a prisoner) requested treatment for a lump on his left testicle that turned out to be a varicocele. He claims that even after repeated visits, his care team did not adequately address his issues. He alleges there were delays in care and diagnosis, as well as ineffective treatment that led to his pain worsening and becoming chronic.	Violation of the 8th Amendment by acting with deliberate indifference to the plaintiff’s serious medical needs.	Chronic pain due to delayed treatment of varicocele	Favoring defense (Wexford Health Sources, Inc.; and Estate of Saleh Obaisi) Defense did NOT include a urologist	Defense provided evidence that they undertook efforts to diagnose and treat the plaintiff’s pain, proving that they were not deliberately indifferent to the issue.
Burns v. Ashraf (2019)	The plaintiff (a prisoner) complained of pain in scrotum that turned out to be a varicocele. He alleges that defendant did not order imaging and other tests and did not refer him to a urologist.	Violation of 8^th^ amendment to receive adequate medical care	Pain and potential infertility due to lack of treatment.	Favoring defense (did NOT include urologist)	Ruled that plaintiff did not sufficiently show that defendants were deliberately indifferent to his pain and varicocele condition. He was, in fact, seen multiple times and prescribed medication. Moreover, disagreements over treatment plan between defendant and plaintiff do not rise to the level of a constitutional violation.
Greer v. Hawaii (2022)	Plaintiff who suffered from polio as a child also had a varicocele for over 50 years and alleges that physicians withheld available treatment over this time period. Once he realized it was treatable, he sought corrective surgery but was given medication instead.	Disability discrimination and violation of his right to equal protection of laws. He claims that physicians misdiagnosed his varicocele and withheld available and appropriate treatment.	Plaintiff states he continues to suffer from the withholding of treatment for his varicocele	Favoring defense (did NOT include urologist)	The 11^th^ Amendment precludes plaintiff’s claims against the State and its officials. The plaintiff’s claims are also unclear to the court.

The most common alleged damages included pain or suffering (80%, 8/10), infertility (30%, 3/10), and loss of testicle (30%, 3/10). The other alleged damages included loss of earnings/income due to incapacitation (10%, 1/10), infection (10%, 1/10), and loss of potential offspring due to infertility caused by unsuccessful varicocele treatment (10%, 1/10).

A urologist was included among the defendants in 6 of the 10 cases. Other defendants included prison wardens, healthcare institutions, prison complexes, other non-urologist physicians (e.g., radiologists, internists, primary care physicians), nurses (RNs and NPs), and pharmacists). The defense in 8 of the 10 cases included multiple defendants, with some involving up to 18 (Diaz v. Spencer).

The verdict in 90% (9/10) of these cases favored the defendant. The lone case which favored the plaintiff occurred in 1951, included a urologist in the defense, and involved a retained drain inside the scrotum which led to infection and ultimately orchiectomy. 100% (6/6) of the cases involving inmates favored the defendant. The justifications for the rulings in favor of the defense oftentimes cited the lack of definitive evidence of deliberate misconduct by the defendant, medical records that contradict the allegations, and/or testimony given by outside physicians in support of the defendant’s actions and reasonings. To give an example, in Canales v. Abramson, the court ruled in favor of the defense; part of their justification reasoned that a difference in opinion over his varicocele treatment between the medical professional and the patient which resulted in a suboptimal outcome for the patient did not constitute deliberate negligence that could be considered malpractice. Furthermore, although the patient suffered from prolonged pain related to his varicocele, there was no evidence that the defense purposely withheld treatment or denied care. In cases such as this, the onus to provide evidence is placed upon the plaintiff, not on the defense.

Looking more specifically at the cases which involved urologists as the defendant, we found that of the 6 cases, 3 involved an inmate as the plaintiff, and 3 involved non-inmates. The cases occurred in 1931, 1951, 1999, 2009, 2013, and 2014. All the non-inmate cases claimed malpractice as the primary breach of duty, as opposed to the breach of the 8^th^ Amendment in the cases involving inmates. Furthermore, only one urologist had undergone fellowship training.

## Discussion

Our study demonstrates that there have been relatively few legal cases on varicoceles that have reached a verdict and the overwhelming majority of these cases of suspected malpractice or negligence were ruled in favor of the defendant.

The fact that more than half of these cases involved inmates may reflect differences in approach or treatment of urologic issues in inmates amongst medical care facilities. Violation of the 8th Amendment, which prohibits cruel and unusual punishment, was a common breach of duty alleged by incarcerated plaintiffs. However, all those cases favored the defense, with the explanation of these decisions often stating that the act of treating the varicocele, even if not successful, was proof that sufficient efforts were made.

From 2017 to 2021, the most common claims revolved around improper treatment (28.5%), failure to diagnose or inappropriate diagnosis (26%), and surgery (24%) [3].

Regarding breaches of duty, it is noteworthy that there was an even split between cases which cited insufficient treatment or lack of surgery, and cases which cited unsuccessful surgery. This 50:50 distribution reflects the distribution of claims in malpractice lawsuits more broadly, in which claims revolving around either improper treatment or surgery were fairly even (28.5% and 24% of all claims from 2017 to 2021, respectively) [3]. Looking more closely at what constituted an unsuccessful operation, one case (Champion v. Bennetts) cited negligence during the operation due to the urologist leaving a “rubber tube” in the scrotum resulting in orchiectomy; another (Nicholas v. Jacobson) cited infection resulting from the operation; another (Cohen v. Cabrini Medical Center) cited continued infertility after varicocelectomy; and two (Diaz v. Spencer, Abdur-Rahiim v. Doe) cited continued pain after surgery. The lone case in which the jury favored the plaintiff (Champion v. Bennetts) occurred in 1951. To summarize, the plaintiff Champion underwent a varicocelectomy performed by the defendant Dr. Bennetts. While concluding the operation, the doctor inserted a rubber drain that, per the case document “was so negligently placed and so carelessly maintained that as a result thereof appellant was forced to undergo” another operation to remove his testicle due to the resulting infection.

Urologists should also take the opportunity to engage in patient education surrounding expectations for varicocele management. Cohen v. Cabrini Medical Center, for example, highlights this importance: the plaintiff, who was the wife of the patient who underwent a varicocelectomy, had assumed that her husband’s operation would result in increased fertility and assure pregnancy. She was never given these assurances, and so her case was dismissed as being too speculative. Although surgery can certainly improve fertility rates [10], patients should be educated that it does not guarantee pregnancy. Likewise, urologists should also remind patients that surgery does not guarantee pain alleviation, the lack of which led to cases such as Abdur-Rahiim v. Doe and Diaz v. Spencer.

Our study does come with limitations. It does not capture cases which settled prior to trial, and so may give an incomplete picture of litigation procedures surrounding varicocele management. Likewise, although Casetext can provide a comprehensive overview of legal cases, different jurisdictions have different reporting requirements. This can result in inconsistent and incomplete data from the cases. Lastly, the Casetext database is concerned with legal matters and does not necessarily provide a complete medical context with which to analyze each case. As such, we were not privy to the medical documents and patient reports which were cited in the cases.

Nonetheless, providers should be reassured that malpractice cases regarding varicoceles are uncommon and that most cases are ruled in favor of the defense. Moreover, it is important that the context and details of legal cases surrounding urologists are examined and shared to improve patient care and guide urologists in managing varicoceles.

## Conclusion

Varicocele may be associated with pain and subfertility which comes with a range of treatment options, from observation to pain management to surgical correction. Urologists are defendants in more malpractice suits than the average physician and litigation may be a concern when determining their management strategy. Our study indicates that cases involving varicoceles are uncommon, and those involving urologists are even more so. Out of the 10 cases, 6 involved a urologist in the defense. The lone case which ruled against the urologist occurred in 1951. This study serves as a reminder that being sued does not automatically indicate medical error.

## Data Availability

The data was generated using Casetext: https://casetext.com.
